# Effect of a Home Bleaching Agent on the Fracture Toughness of Resin Composites, Using Short Rod Design

**Published:** 2014-06

**Authors:** R. Bagheri, M. Fani, AR. Barfi Ghasrodashti, N. Nouri Yadkouri, SM. Mousavi

**Affiliations:** a Dept. of Dental Materials and Biomaterial Research Centre, School of Dentistry, Shiraz University of Medical Sciences, Shiraz, Iran.; b Student Research Committee, School of Dentistry, Shiraz University of Medical Sciences, Shiraz, Iran.

**Keywords:** Fracture toughness, Resin composites, Bleaching agent

## Abstract

**Statement of Problem:** Resin composites are brittle materials and their major shortcomings are manifested in their sensitivity to flaws and defects. Although various mechanical properties of resin composites have been described, few studies are available on assessing the effect of bleaching agents on resin composites using the short rod design.

**Purpose:** To place various resin composites into distilled water at 37°C for 21 days and determine the effect of immersion time in distilled water, with and without exposure to 10% carbamide peroxide by employing short rod design fracture toughness test.

**Materials and Method:** Specimens were prepared from three resin composites; Rok (SDI), Esthet (Dentsply), and Estelite (Tokuyama). For each material, a total of 24 disc-shaped specimens were prepared using a custom-made mould. Specimens were randomly divided into 3 groups of 8 and conditioned in 37°C distilled water for either 24 hours, or 21 days. 21 day specimens were tested both with and without applying bleaching agent; Polanight (SDI). Study group specimens were bleached for 21 days, 2 hours a day. The specimens were loaded using a universal testing machine with a crosshead speed of 0.5 mm per minute. The maximum load at specimen failure was recorded and the K_Ic_ (MPa. M ^0.5^) was calculated.

**Results:** Statistical analysis using two-way ANOVA showed a significant relationship between material and time (*p*< 0.05).Tukey’s test showed that after 24 h of immersion in distilled water, Rok revealed the highest K_Ic_ followed by Esthet and Estelite. The bleaching agent significantly improved the fracture toughness values of Esthet while it decreased that of Estelite.

**Conclusion:** The fracture toughness of the resin composites was affected by the bleaching agent and distilled water. In comparison with Rok and Estelite, fracture toughness of Esthet was increased due to aging and application of bleaching agent.

## Introduction

In recent years, resin composites have been developed to enhance the challenging requirements of esthetic and strength. To improve esthetics, vital teeth bleaching are frequently requested by patients and have been widely used in conjunction with direct and indirect restorations. External bleaching can be applied by the dentist, staff or by the patient at home. Home bleaching agents vary in their viscosity, flavor, and packing. These products contain various concentrations of carbamide peroxide, hydrogen peroxide, or both with a variety of concentrations. The use of carbamide peroxide has become a popular treatment for whitening discolored teeth. Recently, a published review showed that vital tooth bleaching is comparatively safe in terms of potential risk for alteration of dental hard tissue [1].

For restorations to withstand the functional forces, success is achieved by having an adequate cohesive strength and fracture toughness. Fracture toughness (K_Ic_) is an intrinsic characteristic of a material describing its resistance to crack propagation. The lower the K_Ic _is, the higher the clinical probability of restoration failure under load is, because K_Ic_ defines the critical intensity level at which catastrophic failure occurs due to a micro-defect [2]. Margin and bulk fractures are common reasons for replacing direct esthetic restorations [3] and both occurrences are believed to be related to low fracture toughness [4]. Therefore, it would seem logical to measure the K_Ic_ of restorative dental materials to provide clinicians with a parameter that may be used to assist material selection, thereby potentially increasing the restoration lifespan, especially when retention might be compromised or a heavy functional load will be applied to the restoration.

Controversial reports are available in the literature regarding the effect of bleaching agents on the physical and mechanical properties of resin composites. While application of 30-35% hydrogen peroxide did not affect the surface texture of resin composites [5-6], a slight, but statistically significant increase in the surface roughness and porosity of microfilled and hybrid resin composite has been reported attributable to application of 10-16% carbamide peroxide [7-8]. Turker et al. [9] found a reduction in microhardness of resin composites after application of home-bleaching gels while some others found an increase in hardness [10]. Cullen et al. [11] reported that carbamide peroxide had little to no effect on the tensile strength of resin composites.

Water sorption by a resin composite is dependent on the matrix resin, the filler and the properties of the interface between the matrix and filler [12]. The greater the resin content, the more water is absorbed [13]. It has been shown [14] that water sorption not only affects the materials by weakening the matrix but also affects materials by leaching the fillers into the aqueous media which can result in filler/matrix cracking and hydrolytic degradation of the filler surface [15]. On the other hand, the effect of water sorption causes softening of the composite by penetration into the matrix followed by leaching out of unreacted monomer, degradation and leaching of filler components [16].

A number of different mechanical properties of resin composites have been described in various studies using different tests [8-11]. However, limited information are available on the effect of bleaching agents on the fracture toughness of resin composites using short rod design. Short rod design is one of the four test methods of fracture toughness for dental composites in which tensile load applies in a cylinder containing a chevron-shaped notch. The objective of the present study was to place various commercially available resin composites commonly used for direct restorations into distilled water at 37°C and determine: (1) the effect of immersion time, distilled water with and without exposure to 10% carbamide peroxide using a fracture toughness test; (2) if a relationship exists between the mechanical properties and the material composition of the material tested. The null hypothesis is that the storage media and time do not affect mechanical properties; in other words, there is no relationship between the mechanical properties and material composition.

## Materials and Method

**Figure 1:**
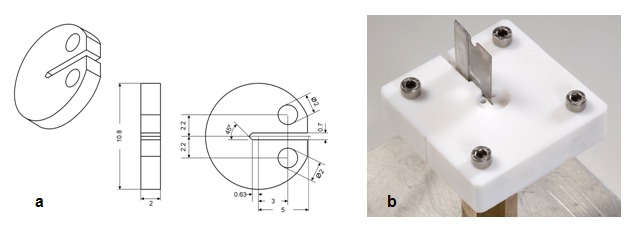
a:  A schematic representation of the specimen configuration along with all dimensions  b: A photo of PTFE split mould assembly with a razor blade placed into the notch during specimen preparation

Three resin composites and one home bleaching agent was used in this in vitro study (Table 1). A custom-made, polytetrafluorethylene (PTFE) split mould was used to prepare a total of 72 disk-shaped specimens (n=8). A pre-crack was created in the mini-compact specimen with a razor blade as described by Kovarik et al. [17] in accordance with ASTM Designation: E399-83 [18]. Figure 1a shows a schematic representation of the specimen configuration along with all dimensions, and Figure 1b displays the PTFE split mould assembly with a razor blade placed into the notch during specimen preparation.

**Table 1:** Materials with technical information

Materials	Manufacturer	Type	Resin	Filler’s type and %	Lot #
Rok	SDI, Vic, Australia	Hybrid composite	UDMA, TEGDMA, Bis-EMA	SAS, AS, (0.04 -2.5 µm); 82.3 Wt% (67.7 Vol %)	120145
Esthet X HD	DENTSPLY, Caulk, USA	Microhybrid composites	BIS-GMA, BIS-EMA, TEGDMA	Barium, FSG (<1 μm), NFS(0.04 μm), TD (nanofiller);(60 Vol%)	1111041
Estelite Sigma Quick	Tokuyama, Dental Co. Tokyo	Submicron filled composite	Bis-GMA, TEGDMA	SiO_2_, ZrO_2_ (200 nm), PFSC (average 0.2 μm); 78Wt% (63 Vol %)	082E61

SAS= Strontium alumino silicate, AS= amorphous silica, FSG= fluoroalumino silicate glass,

NFS= Nanofiller silica, TD= Titanium Dioxide, PFSC= prepolymerized filler of silicacomposite

The split mould was assembled using guide screws. The mould was filled with the resin composite and covered by plastics matrix strips and glass plates under gentle hand pressure in order to extrude excess material. A new razor blade was used to create a sharp pre-crack in the notch during polymerization of the resin composite. The materials were cured according to the manufacturers’ recommended exposure times using an LED curing light with a wavelength range of 440-480 nm at an output of 1500mW/cm^2^(Radii plus LED; SDI, Bayswater, Vic, Australia). Each specimen was removed from the mould and light-cured on the opposite side for an additional period of the recommended exposure time and then placed in distilled water for 30 min. After polymerization, in order to obtain a flat surface, the edges of the specimens were ground by gentle wet grinding using 1500-grit silicon carbide paper.

**Figure 2:**
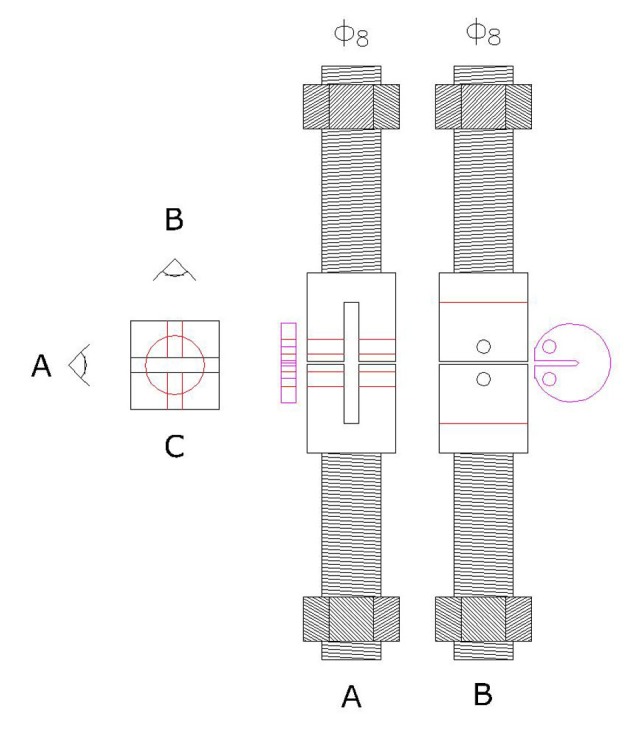
A schematic representation of fracture toughness testing apparatus under tensile loading. Prior to applying load, specimen is secured in Universal testing machine with guide pins placed through specimen’s hole.

The specimens of each material were randomly divided into 3 groups of 8 and stored in distilled water at 37ºC either for 24 h or 21 d. K_Ic_ Baseline measurement was performed after 24 h of immersion. The specimens in the second group were kept in distilled water for 21 d. The specimens in the third group were designated as the treatment group and immersed in a plastic tray fabricated to hold the specimens during the application of bleaching agent, Polanight (SDI; Victoria, Australia), for a period of 21 d, for 2 h a day. Evidently faulty specimens having voids or cracks were discarded. The thickness of each specimen was adjusted to 2 mm (±0.2) and measured using Dental Metal Gauge 04070 (Hygeia Dental Co. Ltd.; Hong Kong, SAR China). The specimens were secured in universal testing machine (Zwick/ Roll Z020; Zwick GmbH & Co, Germany) using guide pins placed through specimens holes (Figure 2).

Tensile loading was applied at a crosshead speed of 0.05 mm/min; the maximum load at specimen failure was recorded and the K_Ic_ (MPa.M^0.5^) was calculated using the following formula:  K_Ic_= Pc× f (a/w) /BW^0.5^

Where, Pc = maximum load at specimen fracture, f (a/w) = a function of a and w   B = specimen thickness, W = dimension from the un-notched edge of the specimen to the plane centreline of the loading holes. 

## Data Analysis

Two-way analysis of variance (ANOVA) was performed to evaluate the interaction of materials and time. Then, one way ANOVA was used for each material using Tukey’s test to compare the effect of distilled water and bleaching agent individually. 

## Results

The results of two-way ANOVA showed a relationship between materials and time (*p*< 0.05). The differences between groups were significant (*p*< 0.02) while the difference within each group was material dependent. Table 2 shows the mean values and standard deviations for K_Ic_ of the tested resin composites in different times and media. After 24 h of immersion in distilled water, Rok revealed the highest K_Ic _(0.75) followed by Esthet X (0.59) and Estelite (0.50) with a significant difference between Rok and other 2 materials and no significant differences between Esthet and Estelite.

**Figure 3:**
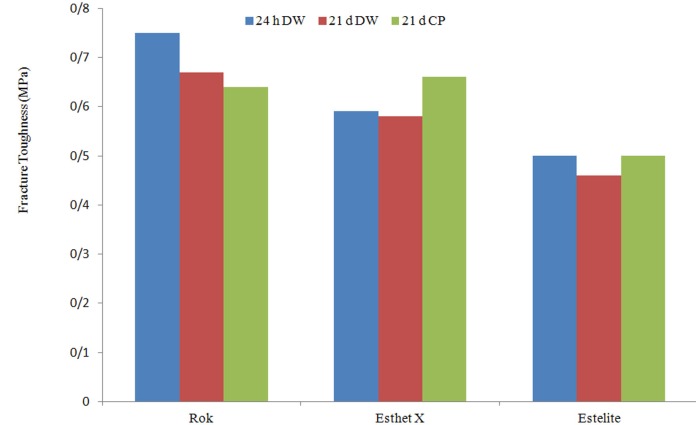
Fracture toughness of all materials including control and treatment groups

Immersion in distilled water for 21 d (Figure 3) was associated with a decrease in the fracture toughness of almost all materials in a significant level for Rok but a slight decrease of Esthet X. Rok was adversely affected by aging in distilled water and even more by carbamide peroxide. A slight decrease was observed for Esthet after aging in distilled water and a significant increase after treatment by carbamide peroxide. Estelite showed a slight decrease after 21 days of immersion in distilled water but no differences in K_Ic_ after treatment by carbamide peroxide was observed (Table 2 and Figure 3).

**Table 2:** Mean fracture toughness (MPa. m^0.5^) and standard deviations (±) of the materials  (n = 8)

**Materials**	**24 Hours Distilled Water**	**21 Days Distilled Water**	**21 Days Carbamide Peroxide**
Rok	^a^0.75 ±(0.07)^A^	^b^0.67 ±(0.07)^A^	^b^0.64 ±(0.08)^A^
Esthet	^a^0.59±(0.05)^B^	^a^0.58±(0.09)^B^	^b^0.66±(0.05)^A^
Estelite	^a^0.50±(0.03)^B^	^b^0.46±(0.03)^C^	^a^0.50±(0.03)^B^

Means with the same upper-case letter in each column are not significantly different (*p*> 0.05).

Means with the same lower-case letter in each row are not significantly different (*p*> 0.05).

## Discussion

The outcome of this study rejected the null hypothesis, indicating that immersion in distilled water with and without applying 10% carbamide peroxide affected fracture toughness of resin composites in different degrees. The effect of bleaching agent on the K_Ic_ (Table 2) varied between materials and was material dependent.

Esthetic restorative materials are marketed in various types with different physical characteristics. One of the main considerations in selecting a resin composite for dental practice is its mechanical properties. Since a restorative material is used to replace the missing tooth structure, resin composite needs to be strong enough to withstand the forces associated with mastication. The success rate of direct resin-composite restorations in the posterior teeth has been found to be 90% over 5 years [19-20].

Due to the wide use of resin composites, it is important to determine which one is more resistant to bulk and marginal fracture. On the other hand, concerning patients demand for lightening the shade of their teeth while having the existing resin composite restoration, it is reasonable to evaluate the effect of whitening agents on the K_Ic _of resin composites. Attin T et al [21] in their study of systematic review on the effect of bleaching on restorative materials found no evidence of negative impact of bleaching on existing restorations.

In the present study, among all resin composites examined, after 24 h of immersion in distilled water, differences between Rok and other two materials were highly significant. Rok showed the highest values while Estelite revealed the lowest (Table 2 and Figure 1b). The differences varied depending on the material composition. The higher baseline values for Rok might be related to the materials’ composition. Compared to Esthet X and Estelite, Rok has the highest filler content (67.7 Vol %) and the largest filler sizes (Table 1). Therefore, Rok’s optimum filler loading and sizes may maximize its fracture resistance. There are controversial reports about the effect of filler size on the fracture toughness of resin composites; one study [17] reported that microfilled resin-composite has optimum fracture toughness, whereas another study [23] showed K_Ic_ values of hybrid and nanofilled composites are significantly higher than those of microfilled composites.

Filler loading has been shown to be in correlation with the material’s strength [24]. Some studies [25-26] have shown that heavier filler loading has a significant impact on the mechanical properties with the highly filled composites being the strongest while others [27] concluded that filler content has no role in fracture behavior.

In addition, the matrix monomers have also a large influence on the strength of different resin composites. Esthet and Estelite with a large proportion of bis-GMA, which has a higher molecular weight, may have an adverse effect on the material’s fracture resistance. Specimens of 21d immersion in distilled water for Rok and Estelite revealed a significant decrease in comparison with the 24 h samples indicating the effect of ageing on the fracture toughness of resin composites. Immersion time has been shown to have an important role in water sorption of resin composites. The degree of water sorption from the oral cavity over time is dependent on the environmental condition and chemical composition of the materials such as matrix resin, filler type and quality of the interface between matrix and filler [28-29]. Hence, water degradation is still considered a problem for resin-based materials.

A recent study comparing the mechanical properties of Esthet and Estelite revealed that Estelite behaved similarly to Esthet in mechanical properties and generalized wear resistance, but exhibited the lower polymerization shrinkage [31].

It is speculated that 15% carbamide peroxide (CP) immediately degrades into one-third hydrogen peroxide (HP) and two-third urea when contacting the tissues and saliva [32]. The authors suggested that peroxides may induce oxidative cleavage of polymer-chains. Thus, unreacted double bonds are vulnerable parts of the polymers [32]. Furthermore, free radicals induced by peroxides may impact the resin-filler-interface and cause debonding of this interface. Due to this effect, different loading of the filler in the materials tested in this study may account for the different effects of the bleaching agents observed in the current study [33].

Home bleaching, supervised by a dentist, is done by the patient at home using custom-made plastic trays that hold the bleaching agent against the patient’s teeth for a period of 2 h a day for 14 or 21 days. The effect of external bleaching lasts approximately six months to two years, after which the teeth often return to their original color [34]. The concentration of CP used in the current experiment was 10% and the period of 21 days of immersion in distilled water in this work was chosen as a control group for 21 day treatment by the bleaching agent. Applying CP for a period of 21 days was associated with increasing K_Ic_ of Esthet X and decreasing that of Rok, with no noticeable changes on Estelite. Similar reports have been published by Cho et al. [35]. In their study on determining the effect of bleaching agents on the fracture toughness of four nano-filled resin composites using 3-point bending test, reported that bleaching had a significant increasing effect on the fracture toughness value of some of the materials [35]. Surface roughness alteration has been shown to be material-related and time-dependent. The bleaching gels affected surface of nanofilled and microhybrid composite resins more than enamel surfaces [36].

However, performance of Esthet in our study is different from the results of Cho et al. [35] who reported that after 24 h (control group), K_Ic _value of EsthetX was significantly higher than that of other composites. The application of bleaching agents did not significantly change the fracture toughness values of Esthet X, while significantly improved that of Filtek Supreme. The differences may be due to the way in which the degrading compound was applied to the material surface. These differences are also a good indication of the effect of geometry and methodology on the measurement of fracture toughness of resin composites. Specimen shape and stress concentration during tests in two different methods seem to have a direct effect on the results.

In addition, Rok and Estelite lost their strength after 21 days of immersion in distilled water. One possible reason for this could be their ability for water sorption. It has been reported that water sorption is influenced by the quantity of hydroxyl groups within the matrix, and the creation of hydrogen bonds with water [37].These two composites have a high proportion of TEGDMA which has been shown to exhibit higher diffusion coefficients than resins such as U/EDMA [38].

As shown in Table 2 and graphically in Figure 3, Rok with the highest value at base line lost its toughness remarkably after 21 days of immersion in distilled water and even more with applying 10% carbamide peroxide.In contrast, Esthet X with the lower baseline value showed an improved toughness after ageing and Estelite did not lose its strength. Therefore, it is speculated that it may be practical for clinicians to use those composites for which their strength remains more stable over time and in different media.

## Conclusion

The fracture toughness of the resin composites was affected by the bleaching agent and distilled water. In comparison with Rok and Estelite, fracture toughness of Esthet was increased due to aging and application of bleaching agent. The results of this study suggest that it may be prudent for clinicians to use those resin composites for which their strength remains more stable over time especially when bleaching treatment might be a future possibility. Indeed, it may not be beneficial to use bleaching agent for those patients who have many composite fillings in their mouth. It is important to emphasize that the results of the present study are valid for the laboratory conditions used. Therefore, the results of this study must be interpreted with caution since the clinical performance of resin composites in conjunction with bleaching agent may differ due to the complex nature of the oral cavity. 
